# Sexual function and depressive symptoms in young women with elevated macroprolactin content: a pilot study

**DOI:** 10.1007/s12020-016-0898-5

**Published:** 2016-02-22

**Authors:** Robert Krysiak, Agnieszka Drosdzol-Cop, Violetta Skrzypulec-Plinta, Bogusław Okopien

**Affiliations:** Department of Internal Medicine and Clinical Pharmacology, Medical University of Silesia, ul. Medyków 18, 40-752 Katowice, Poland; Chair of Woman’s Health, School of Health Sciences in Katowice, Medical University of Silesia, ul. Medyków 12, 40-752 Katowice, Poland

**Keywords:** Depressive symptoms, Hyperprolactinemia, Macroprolactin, Polyethylene glycol assay, Prolactin, Sexual functioning

## Abstract

Elevated prolactin levels seem to be associated with impaired sexuality. The clinical significance of macroprolactinemia, associated with the predominance of high molecular mass circulating forms of prolactin, is still poorly understood. This study was aimed at investigating sexual function in young women with macroprolactinemia. The study enrolled 14 young women with macroprolactinemia, 14 with increased monomeric prolactin levels, as well as 14 age- and weight-matched healthy women. All patients completed a questionnaire evaluating female sexual function (Female Sexual Function Index—FSFI), as well as a questionnaire assessing the presence and severity of depressive symptoms (Beck Depression Inventory Second Edition—BDI-II). Apart from total prolactin levels and macroprolactin content, circulating levels of thyrotropin, total testosterone, and 17-β estradiol were also measured. Patients with elevated monomeric prolactin levels had a lower total FSFI score, as well as lower scores for all domains: sexual desire, sexual arousal, lubrication, orgasm, sexual satisfaction, and dyspareunia. These scores correlated with total and monomeric prolactin levels. In turn, women with macroprolactinemia were characterized by a lower score for sexual desire, and only this score correlated with total prolactin levels and macroprolactin content. The total score in the BDI-II questionnaire was higher in patients with hyper- and macroprolactinemia than in the control subjects. Contrary to multidimensional impairment of sexual function in women with elevated monomeric prolactin, macroprolactinemia only seems to disturb sexual desire.

## Introduction

The most common symptoms of prolactin excess in premenopausal women are oligomenorrhea/amenorrhea and galactorrhea. This results from the inhibitory effect of prolactin on gonadotropin-releasing hormone secretion in the hypothalamus and from a stimulatory effect of high concentrations of this hormone on the proliferation and differentiation of mammary cells playing a role in lactation [1, 2]. It seems that this hormone may be involved in the regulation of sexual functioning. An increase in prolactin levels is observed in healthy men and women following an orgasm induced by both masturbation or coitus, but not after sexual arousal without orgasm [3, 4]. The magnitude of the intercourse post-orgasmic prolactin surge correlates with both orgasm quality and subsequent sexual satisfaction [5]. Penile-vaginal intercourse produces a substantially greater post-orgasmic prolactin increase than masturbation does [6]. These findings indicate that post-orgasmic prolactin surges may be a neurohormonal index of sexual satiety as well as an objective index of orgasm and orgasm quality [5, 6]. Acute prolactin increases following an orgasm are involved in a negative feedback loop that serves to decrease arousal through inhibitory central dopaminergic and probably peripheral processes [3, 6]. Cabergoline, a drug strongly reducing prolactin levels, was found to enhance all parameters of sexual drive, function, and positive perception of the refractory period [7]. In turn, protirelin, a stimulator of prolactin secretion, produced significantly longer ejaculation latency, and reduced insignificantly sexual drive and function [7]. Men with hyperprolactinemia, induced by prolactin-secreting tumors, were characterized by a higher prevalence of hypoactive sexual desire than matched healthy men [8]. A relevant hypoactive sexual desire was observed in the minority of patients with severe hyperprolactinemia, among a group of patients with erectile dysfunction [9]. However, in another study [10], hyperprolactinemia did not affect night erections or penile response to visual erotic stimuli. Most women (like most men) with elevated prolactin levels and well-defined hypothalamo-pituitary disease developed a lack of or a marked decrease in sexual desire, and many of them complained of problems with lubrication or orgasm [11]. Kadioglu et al. [12], using the Female Sexual Function Index (FSFI) questionnaire, observed statistically lower values of the total FSFI score and domain scores for all phases of female sexual function: desire, arousal, lubrication, orgasm, satisfaction, and pain in females with elevated prolactin levels, and this sexual dysfunction correlated negatively with circulating prolactin levels.

Based on molecular mass, prolactin can be divided into monomeric prolactin (23 kDa), big prolactin (45–50 kDa), and macroprolactin (or big–big prolactin) (150–170 kDa). Consisting mainly from complexes of prolactin with IgG, macroprolactin may be also composed of complexes formed by prolactin and IgA or IgM, or aggregates of covalent or non-covalent polymers of monomeric prolactin [13–15]. The term “macroprolactinemia” is used when macroprolactin content exceeds 60 % of the total circulating levels determined by polyethylene glycol precipitation [16, 17]. Although most subjects are asymptomatic, in the remaining ones, macroprolactinemia may result in galactorrhea, oligomenorrhea/amenorrhea, subfertility/infertility, and gynecomastia [18–20]. Sexual disturbances were previously observed in a few studies involving men with elevated levels of big–big prolactin. Erectile dysfunction, as the most frequently described complication, was found in 50 % [21] or 78 % [22] of macroprolactinemic men, and its frequency was similar to that observed in males with monomeric hyperprolactinemia [23, 24]. Moreover, some patients complained of diminished libido and infertility [21, 22].

To the best of our knowledge, no study investigated sexual function in women with macroprolactinemia. Therefore, the aim of the present study was to compare sexual function and depressive symptoms between women with elevated levels of monomeric and big–big prolactin.

## Materials and methods

### Study population

The participants of the study (young women aged 20–40 years) were recruited on the basis of personal history of hyperprolactinemia, oligomenorrhea, galactorrhea, or traumatic brain injury, women with incidentally found pituitary microadenomas or those planning pregnancy. These individuals, initially supervised by community-based health care providers, were screened in the Chair of Woman’s Health for the presence of endocrine abnormalities. If serum prolactin levels exceeded 30 ng/mL, the measurement was repeated together with the measurement of macroprolactin content. The study population included only women with elevated prolactin levels (above 30 ng/mL) found during both of these measurements. The participants were divided into two groups: women with true hyperprolactinemia (*n* = 14) and women with macroprolactinemia (*n* = 14). The study also enrolled 14 age- and weight-matched healthy women. This sample size was based on a power analysis conducted before the study, which considered our previous results [25]. Considering a power of 80 % and a significance (α) level of 0.05, at least 14 women in each treatment group would need to be included to detect a 15 % difference in the overall FSFI score. We excluded patients with mixed pituitary tumors (secreting prolactin and other pituitary hormones), other hormonally active and non-functioning pituitary microadenomas, pituitary macroadenomas, impaired renal or hepatic function, thyroid disorders, polycystic ovary syndrome, any acute or chronic disease, psychiatric problems, postpartum complications, developmental or acquired anomalies of the female reproductive system and who underwent urogynecological operations that might affect sexual function, as well as pregnant, breastfeeding, and sexually inactive women.

The study was approved by the Bioethics Committee of the Medical University of Silesia in Katowice, Poland. All enrolled patients provided their written informed consent for the investigation.

### Methods

Venous blood samples were collected from patients while in their early follicular phase (days 2 and 5, counting from the first day of the last menstrual period) between 8.00 and 9.00 a.m., after a 12-h overnight fast, in a quiet, temperature-controlled room (24–25 °C). Samples from two women with monomeric hyperprolactinemia, who developed secondary amenorrhea, were collected irrespectively of the menstrual cycle phase. To minimize analytical errors, all assays were conducted in duplicate by a person unaware of the patient’s data. Serum levels of prolactin were measured before and shortly after polyethylene glycol precipitation, using reagents from DRG Instruments GmbH (Marburg, Germany). Serum levels of total testosterone and 17β-estradiol were determined by an enzyme-linked immunosorbent assay (DRG Instruments GmbH, Marburg, Germany), while serum levels of thyrotropin were measured using an electrochemiluminescence immunoassay method (Roche Diagnostics, Lewes, United Kingdom). Macroprolactin content was further assessed on the basis of results obtained from serum prolactin levels [26]. Equal volumes (250 μL) of sera and 25 % cold polyethylene glycol 6000 dissolved in phosphate-buffered saline (Sigma, 137 mmol/L sodium chloride, 10 mmol/L sodium phosphate, pH = 7.4) were mixed in the ratio of 1:1 and incubated for 10 min. After vortex mixing for 30 s, the suspension was clarified by centrifugation at 3000×*g* for 30 min, before prolactin measurement. To correct for the dilution with polyethylene glycol, the post-polyethylene glycol prolactin concentration was determined by multiplying the prolactin result by 2. Macroprolactinemia was diagnosed if the prolactin recovery was <40 %.

Sexual function and depressive symptoms were assessed in all women considered for enrollment immediately after second blood collection. Neither the individuals nor the investigators were aware of the prolactin status of the patient. The participants were asked to complete a questionnaire evaluating their sociodemographic characteristics (age, marital status, education, occupational activity, type of work, profession, physical activity, stress exposure), medical history, obstetric and gynecological history.

Women’s sexual function in the last 4 weeks was evaluated using FSFI. This survey consists of 19 questions, divided into 6 domains: desire (items 1 and 2), arousal (items 3–6), lubrication (items 7–10), orgasm (items 11–13), satisfaction (items 14–16), and pain (items 17–19) [27, 28]. Each answer is rated on a scale ranging from 0 to 5 or 1 to 5, with 0 indicating no sexual activity in the past 4 weeks. The overall score, being the sum of the scores for each item multiplied by a domain factor (0.6 for desire, 0.3 for arousal and lubrication, and 0.4 for orgasm, satisfaction, and pain), may range from 2 to 36, with lower values indicating higher symptom burden. Women with the overall less than 26.55 are classified as presenting sexual dysfunction [27, 28].

Depression severity was measured with the Beck Depression Inventory Second Edition (BDI-II), being a valid and reliable measure of depressive state [29], which corresponds well to a clinical diagnosis of depressive disorders described in the Diagnostic and Statistical Manual of Mental Disorders, Fourth Edition [30]. BDI-II consists of 21 items rated on a scale from 0 to 3. The BDI-II score is interpreted according to manual guidelines [29]: minimal range 0–13, mild depression = 14–19, moderate depression = 20–28, and severe depression = 29–63.

### Statistical analysis

Although all potential participants underwent laboratory investigations and completed the questionnaires, only data of patients who met the inclusion and exclusion criteria, as well as data of control subjects with normal prolactin levels, were included in the final analyses. The normality of distribution was determined using the Kolmogorov–Smirnov test. Due to the skewed distributions, quantitative values were natural-log transformed to satisfy assumptions of normality and equal variance. Comparisons between the groups were performed using analysis of covariance followed by Bonferroni post hoc tests after consideration of age, smoking, body mass index, waist circumference, marital status, education, occupational activity, type of work, profession, physical activity, stress exposure, as well as well as blood pressure as potential confounders. The *χ*^2^ test was used for all qualitative variables. Correlations were assessed using Pearson’s test. Statistical significance was assumed at *p* < 0.05.

## Results

### General characteristics of the study groups

**Fig. 1:**
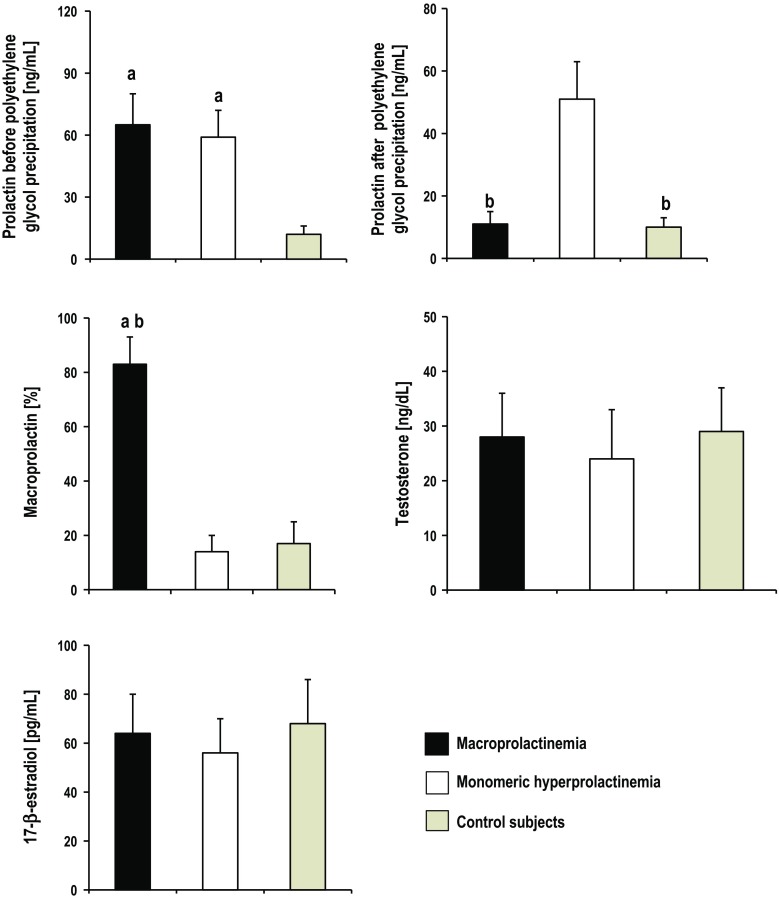
Serum levels of the investigated markers. All means and standard deviations represent unadjusted values. ^1^[1- (serum prolactin after polyethylene glycol precipitation/serum prolactin before polyethylene glycol precipitation)] × 100. ^a^ *p* < 0.001 versus control women; ^b^ *p* < 0.001 versus women with monomeric hyperprolactinemia

The characteristics of the included patients are summarized in Table 1. There were no significant differences between the groups among variables of age, body mass index, waist circumference, smoking (pack-years of smoking), education, occupational activity, type of work performed, stress exposure, number of abortions, as well as systolic and diastolic blood pressure. Differences were observed between the study groups with relation to physical activity, number of sexual partners, number and duration of marriages, as well as number of deliveries. As expected, women with hyperprolactinemia and macroprolactinemia had higher circulating levels of total prolactin than healthy subjects, with no difference between both groups (Fig. 1). Prolactin, after polyethylene glycol precipitation was higher in patients with hyperprolactinemia than in women with macroprolactinemia and women with normal total prolactin concentrations. Macroprolactin content was higher in patients with macroprolactinemia than in the remaining groups of patients (Fig. 1). Moreover, women with elevated levels of monomeric prolactin have insignificantly lower levels of testosterone (*p* = 0.075) and 17β-estradiol (*p* = 0.086) than healthy subjects (Table 1, Fig. 1).

**Table 1:** Sociodemographic characteristics and serum hormone levels in the study population

	Macroprolactinemia	Monomeric hyperprolactinemia	Control subjects
Number of patients	14	14	14
Age [years; mean (SD)]	31 (4)	30 (5)	29 (5)
Body mass index [kg/m^2^; mean (SD)]	25.7 (2.9)	26.2 (3.7)	24.9 (3.4)
Waist circumference [cm; mean (SD)]	87 (11)	88 (13)	86 (10)
Smokers (%)/pack-years of smoking [mean (SD)]	21/3.13 (0.63)	21/3.76 (0.73)	21/4.3 (0.95)
Physical activity: total/once a week/several times a week/once a month (%)	57/14/21/21*	57/21/14/21*	79/29/29/21
Primary or vocational/secondary/university education (%)	36/36/29	36/29/36	29/36/36
Occupational activity/blue-collar/white-collar/pink-collar workers (%)	71/21/29/21	71/29/21/21	79/29/29/21
Number of sexual partners [*n*; mean (SD)]	1.7 (0.5)*	1.6 (0.7)*	2.2 (0.7)
Number of marriages [*n*; mean (SD)]/duration of marriages [months; mean (SD)]	1.1 (0.5)/58 (14)	1.0 (0.5)*/40 (13)*	1.3 (0.5)/55 (16)
Number of deliveries [n; mean (SD)]/number of abortions [*n*; mean (SD)]	1.0 (0.5)/0.2(0.4)	0.7 (0.6)*/0.3(0.4)	1.2 (0.5)/0.2 (0.4)
Stress exposure [%, mean (SD)]	79	71	71
Systolic blood pressure [mm Hg; mean (SD)]	117 (9)	121 (11)	115 (10)
Diastolic blood pressure [mm Hg; mean (SD)]	74 (5)	76 (5)	73 (6)
Prolactin before polyethylene glycol precipitation [ng/mL]^b^	65 (15)**	59 (13)**	12 (4)
Prolactin after polyethylene glycol precipitation [ng/mL]^c^	11 (4)***	51 (12)	10 (3)***
Macroprolactin (%)^a,d^	83 (10)**,***	14 (6)	17 (8)
Testosterone [ng/dL; mean (SD)]	28 (8)	24 (9)	30 (8)
17β-estradiol [pg/mL; mean (SD)]	64 (16)	56 (14)	68 (18)

All means and standard deviations (SD) represent unadjusted values

* *p* < 0.05, ** *p* < 0.001 versus control women; *** *p* < 0.001 versus women with monomeric hyperprolactinemia

^a^[1 − (serum prolactin after polyethylene glycol precipitation/serum prolactin before polyethylene glycol precipitation)] × 100

^b^ *F*[2,39] = 38.6, *p* < 0.001

^c^ *F*[2,39] = 41.3, *p* < 0.001

^d^ *F*[2,39] = 46.2, *p* < 0.001

### Assessment of sexual function

Women with hyperprolactinemia, excluding those with macroprolactinemia, had lower total scores on the FSFI than control subjects (Table 2). The total FSFI score not exceeding 26.55 was found among 7 (50 %) women with monomeric hyperprolactinemia, 4 (29 %) with macroprolactinemia, and in 2 (14 %) patients belonging to the control group. Subjects with elevated levels of monomeric prolactin obtained lower scores in all six domains (sexual desire, sexual arousal, lubrication, orgasm, sexual satisfaction, and dyspareunia), while women with macroprolactinemia only in one domain (sexual desire). There were differences between macroprolactinemia and monomeric prolactin in lubrication and orgasm (Table 2).

**Table 2:** Female sexual function in women with macroprolactinemia, monomeric hyperprolactinemia, and normal prolactin levels

Variable	Macroprolactinemia	Monomeric hyperprolactinemia	Control subjects
FSFI score [mean (SD)]^a^	30.05 (2.85)	27.00 (3.10)*^,††^	31.67 (3.15)
FSFI score ≤ 26.55 (%)	29*	50**^,†^	14
Sexual desire [mean (SD)]^b^	4.21 (0.60)**	4.01 (0.64)**	5.00 (0.52)
Sexual arousal [mean (SD)]^c^	5.36 (0.44)	4.88 (0.53)*	5.42 (0.55)
Lubrication [mean (SD)]^d^	5.31 (0.41)	4.53 (0.68)**^,††^	5.50 (0.42)
Orgasm [mean (SD)]^e^	4.92 (0.53)	4.41 (0.51)**^,†^	5.10 (0.63)
Sexual satisfaction [mean (SD)]^f^	5.03 (0.62)	4.45 (0.65)*	5.28 (0.64)
Dyspareunia [mean (SD)]^g^	5.22 (0.53)	4.72 (0.58)*	5.37 (0.53)

All means and standard deviations (SD) represent unadjusted values

* *p* < 0.05, ** *p* < 0.01, ** *p* < 0.001 versus control subjects; ^†^ *p* < 0.05, ^††^ *p* < 0.01 versus women with macroprolactinemia

^a^ *F*[2,39] = 5.2, *p* < 0.01

^b^ *F*[2,39] = 7.1, *p* < 0.01

^c^ *F*[2,39] = 3.4, *p* < 0.05

^d^ *F*[2,39] = 8.7, *p* < 0.01

^e^ *F*[2,39] = 4.9, *p* < 0.05

^f^ *F*[2,39] = 4.2, *p* < 0.05

^g^ *F*[2,39] = 3.5, *p* < 0.001

In women with monomeric hyperprolactinemia, sexual arousal, lubrication, orgasm, sexual satisfaction, and dyspareunia inversely correlated with total prolactin levels and prolactin concentration after polyethylene glycol precipitation (*r* values between −0.25, *p* < 0.05 and −0.41, *p* < 0.001).

In women with macroprolactinemia, there were inverse correlations between sexual desire and total prolactin concentration (*r* = −0.24, *p* < 0.05) or macroprolactin content (*r* = −0.35, *p* < 0.01). No other correlations were found.

### Assessment of depressive symptoms

The total BDI-II score was higher, while total and mild depressive symptoms occurred more frequently in women with macroprolactinemia or monomeric hyperprolactinemia than in control subjects. There were no significant differences in the total BDI-II score, as well as in the number of patients experiencing total and mild depressive symptoms between women with elevated levels of big–big prolactin and females with elevated levels of monomeric prolactin (Table 3).

**Table 3:** Depressive symptoms in women with macroprolactinemia, monomeric hyperprolactinemia, and normal prolactin levels

Variable	Macroprolactinemia	Monomeric hyperprolactinemia	Control subjects
BDI-II score [mean (SD)]^a^	11.9 (3.1)*	12.9 (3.5)*	8.0 (3.7)
Depressive symptoms [*n* (%)]	6 (43)*	7 (50)*	3 (21)
Mild symptoms [*n* (%)]	6 (43)*	6 (43)*	3 (21)
Moderate symptoms [*n* (%)]	0 (0)	1 (7)	0 (0)
Severe symptoms [*n* (%)]	0 (0)	0 (0)	0 (0)

All means and standard deviations (SD) represent unadjusted values

^*^ *p* < 0.05 versus control subjects

^a^ *F*[2,39] = 5.3, *p* < 0.01

In women with elevated total prolactin levels, the mean total FSFI score inversely correlated with the total BDI-II score (women with macroprolactinemia: *r* = −0.26, *p* < 0.05; women with monomeric hyperprolactinemia: *r* = −0.41, *p* < 0.001).

## Discussion

This report shows for the first time that macroprolactinemia is associated with impaired sexual desire but not with the changes in other domains of the FSFI. In light of the results of studies involving men [21–24], the present findings suggest that sexual disturbances in macroprolactinemic patients are not limited to only one gender. It seems that macroprolactinemia should be taken into account in all cases of hyperprolactinemia, particularly if it coexists with dysfunction of only the first phase of sexual response cycle.

Having a large molecular size, macroprolactin does not cross the endothelial lining and therefore it probably lacks biological activity in vivo [31]. Impaired sexual desire observed in macroprolactinemic women is in some disagreement with this finding. Our results rather suggest that either big–big prolactin stimulates prolactin receptors, or, as hypothesized by Gibney et al. [18], particularly in the cases in which prolactin creates complexes with low-affinity, high-capacity IgG, macroprolactin dissociates, releasing monomeric prolactin. Owing to its high molecular weight, macroprolactin has substantially longer renal clearance than monomeric prolactin [14, 15], and as a result of this, it accumulates in the serum and probably also in tissues. Therefore, it may stimulate prolactin receptors weakly, but persistently.

In line with the latter hypothesis, impaired desire, the only sexual dysfunction observed in macroprolactinemic women, was the most frequently affected domain in our group of patients with monomeric hyperprolactinemia, as well as in hyperprolactinemic women assessed by Kadioglu et al. [12]. It may be assumed that the amount of monomeric prolactin released locally from macroprolactin complexes is lower than that found in the sera of patients with hyperprolactinemia. In line with the latter explanation, only desire, but not other subscales of women’s sexual function assessed in our study, correlated with big–big prolactin content.

To the best of our knowledge, only two groups of agents were found to affect macroprolactin content. Dopamine reduced macroprolactin in selected macroprolactinemic patients [21, 32], while the opposite effect was induced by oral contraceptive pills containing ethinylestradiol and levonorgestrel [26]. The possible association between macroprolactinemia and impaired sexual function, and previously mentioned concerns about the possible relationship between macroprolactinemia and menstrual abnormalities and galactorrhea [18] suggest that in women in whom elevated levels of macroprolactin coexists with monomeric hyperprolactinemia, dopamine receptor agonists should be preferred to oral contraceptive pills, even if they do not desire pregnancy.

Sexual desire, sexual arousal, lubrication, orgasm, sexual satisfaction, and dyspareunia in subjects with monomeric hyperprolactinemia correlated with circulating levels of pre- and post-polyethylene glycol precipitation prolactin. The presence of correlations between all domain scores and monomeric prolactin levels suggests that the degree of hyperprolactinemia, not the underlying disorder, is the main factor determining the severity of sexual dysfunction. Interestingly, despite slight differences in serum levels of testosterone and 17β-estradiol between patients with monomeric hyperprolactinemia and healthy controls, sexual function did not depend on serum concentrations of sex hormones. Our findings indicate that sexual disturbances in hyperprolactinemic patients are probably secondary to the central effects of prolactin and, at least in the investigated group of women, cannot be explained by low testosterone and 17β-estradiol levels. Total prolactin levels in our study were moderately elevated and this finding explains why testosterone and 17β-estradiol levels were only insignificantly decreased. It cannot be excluded that secondary hypogonadism may contribute to sexual abnormalities in patients with severe monomeric hyperprolactinemia. Low thyroid hormone levels, increasing thyrotropin-releasing hormone secretion, stimulates lactotrophs to produce prolactin [33, 34], while hypothyroidism was found to induce sexual dysfunction [35]. However, normal thyrotropin levels allow us to exclude the impact of hypothyroidism.

Depressive symptoms experienced by patients with monomeric hyperprolactinemia was an expected finding in our study, because similar relationships were observed previously by other research groups, and may be, at least in part, interpreted by the impact of the hormone excess on dopaminergic and/or serotoninergic transmission in the central nervous system [36–38]. More importantly, reduced desire observed in women with macroprolactinemia was also accompanied by an increased total BDI-II score. The association between low libido and the BDI-II score indicates that either depressive symptoms impair sexual desire or impaired desire contributes to the development of depressive symptoms in the investigated group of women. Because the correlation between desire and depressive symptoms was weak, it seems that the latter may result additionally from other directions of action of big–big prolactin.

We are aware of some limitations of this study. Firstly, the study sample is too small to generalize the obtained results. Secondly, as in other self-report inventories, FSFI and BDI-II scoring systems are subjective in nature. Thirdly, another shortcoming of the FSFI is the explicit failure to differentiate between sexual behaviors. Fourthly, macroprolactin content was determined using a precipitation method and the results of our study should be confirmed by gel filtration chromatography, which is the gold standard technique for the diagnosis of macroprolactinemia [16]. Finally, because the study excluded patients receiving pharmacological treatment, it cannot be excluded that sexual function may be different in macroprolactinemic patients receiving pharmacotherapy.

In conclusion, young women with macroprolactinemia reported disturbances in only one domain of female sexual function, which was much less expressed than in women with elevated monomeric prolactin levels. Our study suggests that reduced sexual desire in hyperprolactinemic women, not accompanied by other sexual complications, justifies their assessment for the presence of macroprolactin.
